# Overtreatment in the United States

**DOI:** 10.1371/journal.pone.0181970

**Published:** 2017-09-06

**Authors:** Heather Lyu, Tim Xu, Daniel Brotman, Brandan Mayer-Blackwell, Michol Cooper, Michael Daniel, Elizabeth C. Wick, Vikas Saini, Shannon Brownlee, Martin A. Makary

**Affiliations:** 1 Department of Surgery, Brigham and Women’s Hospital, Harvard Medical School, Boston, Massachusetts, United States of America; 2 Department of Surgery and the Department of Medicine, Johns Hopkins University School of Medicine, Baltimore, Maryland, United States of America; 3 The Lown Institute, Boston, Massachusetts, United States of America; 4 Department of the Department of Health Policy and Management, Johns Hopkins Bloomberg School of Public Health, Baltimore, Maryland, United States of America; University of Miami, UNITED STATES

## Abstract

**Background:**

Overtreatment is a cause of preventable harm and waste in health care. Little is known about clinician perspectives on the problem. In this study, physicians were surveyed on the prevalence, causes, and implications of overtreatment.

**Methods:**

2,106 physicians from an online community composed of doctors from the American Medical Association (AMA) masterfile participated in a survey. The survey inquired about the extent of overutilization, as well as causes, solutions, and implications for health care. Main outcome measures included: percentage of unnecessary medical care, most commonly cited reasons of overtreatment, potential solutions, and responses regarding association of profit and overtreatment.

**Findings:**

The response rate was 70.1%. Physicians reported that an interpolated median of 20.6% of overall medical care was unnecessary, including 22.0% of prescription medications, 24.9% of tests, and 11.1% of procedures. The most common cited reasons for overtreatment were fear of malpractice (84.7%), patient pressure/request (59.0%), and difficulty accessing medical records (38.2%). Potential solutions identified were training residents on appropriateness criteria (55.2%), easy access to outside health records (52.0%), and more practice guidelines (51.5%). Most respondents (70.8%) believed that physicians are more likely to perform unnecessary procedures when they profit from them. Most respondents believed that de-emphasizing fee-for-service physician compensation would reduce health care utilization and costs.

**Conclusion:**

From the physician perspective, overtreatment is common. Efforts to address the problem should consider the causes and solutions offered by physicians.

## Introduction

Waste in health care is increasingly being recognized as a cause of patient harm and excess costs. In 2010, the Institute of Medicine (IOM) called attention to the problem, suggesting that “unnecessary services” are the largest contributor to waste in United States (US) health care, accounting for approximately $210 billion of the estimated $750 billion in excess spending each year.[1] The report estimated the cost using four analytically distinct studies, including two projections of excess spending associated with variation in care.[2, 3] In previous studies of specialty care, it has been observed that 30% of inpatient antimicrobial therapy,[4] 26% of advanced imaging,[5] and 12% of acute percutaneous coronary interventions are unnecessary or inappropriate.[6] Reducing overtreatment has important implications for improving patient-centered care.[7] Overtreatment is directly associated with patient harm as evidenced by studies of antibiotic overuse leading to resistance and *Clostridium difficile* infection,[8] overuse of diagnostic testing, such as pap smear and colonoscopy,[9, 10] and the inherent postoperative complications from unnecessary surgical procedures.[11]

To date, however, direct estimates by physicians of the prevalence of unnecessary care have been limited. There was one survey study conducted in 2009 by Sirovich et al that studied primary care physician perceptions on overall utilization of health care resources.[12] Clinical appropriateness remains difficult to measure, and studies relying on large datasets or chart review rarely capture pivotal details of a patient’s clinical presentation and are subject to reporting bias. Furthermore, thresholds to intervene medically can be soft and influenced by a lack of awareness about best practices or by perverse financial incentives, which have been implicated as a contributor to unnecessary care.[13, 14] Physicians have a unique front-line perspective on the problem of overtreatment given their direct role in recommending, managing, and observing tests, procedures, and medications. To draw on this knowledge, we designed a study to estimate physicians’ perspective on the prevalence of overtreatment in health care and identify potential causes and solutions.

## Materials and methods

All medical doctors listed in the American Medical Association (AMA) master file were previously invited on an annual basis to participate in an online educational community (QuantiaMD) which includes 160,000 U.S. physicians, representing 28% of U.S. physicians. Members of this community were invited by email between January 22, 2014 and March 8, 2014 to complete a survey about physician practice patterns. Participation was voluntary, and each participant was paid a $5 gift voucher for completing the survey. Financial resources to provide thank you gift cards to doctors who completed the survey determined how many were invited. We randomly selected 3,318 doctors from the educational community of 160,000 doctors and 2,327 clinicians completed the survey, for a 70.1% survey response rate. We excluded 213 clinicians who we identified to be midlevel clinicians and 8 physician respondents who did not complete the last three questions, yielding a final study population of 2,106 physicians. Mid-level providers included physician assistants and nurse practitioners; they were excluded for the purposes of keeping the survey respondents a uniform population of physicians. After excluding midlevel providers and 8 respondents that did not complete the survey, we had a final study population representing 63.5% of invitees. General Internal Medicine, Pediatrics, and Family Medicine were considered primary care, while all other specialties were considered specialist care. Specialized Internal Medicine specialties included Allergy and Immunology, Cardiology, Endocrinology, Gastroenterology, Geriatrics, Hematology and Oncology, Infectious Disease, Interventional Cardiology, Nephrology, Pulmonary and Critical Care, Rheumatology, and Sleep Medicine. The distribution of physicians by specialty in the study population (Table 1) relative to the distribution of physicians in the U.S. was skewed toward primary care but still included a high number of specialists (57.6% vs. 33.4% primary care, 42.4% vs. 66.6% specialists, respectively).[15]

**Table 1:** Respondent characteristics (N = 2,106).

Clinician characteristics		%
Male	1368	65.0%
Experience		
Trainee	1097	52.1%
Attending, less than 10 years	545	25.9%
Attending, 10 or more years	464	22.0%
Specialty		
Primary care	1213	57.6%
Specialist	893	42.4%
Type of compensation		
Salary only	1358	64.5%
Fee-for-service	746	35.4%
**Hospital Characteristics**		
Hospital size		
<250 beds	409	19.4%
250–500 beds	835	39.6%
>500 beds	862	40.9%
Hospital type		
VA/Government*	220	10.4%
For-profit	484	23.0%
Non-profit	1402	66.6%
Type of institution		
Academic	1565	74.3%
Non-academic	541	25.7%
Setting		
Urban	1423	67.6%
Suburban	535	25.4%
Rural	148	7.0%

*integrated health care system serving American veterans

The survey queried physician attitudes on overtreatment, and the causes and solutions for the problem. The survey was piloted to six focus groups over a 9-month period prior to the study period. Focus groups were qualitative, asking physicians 3 questions after completing the survey: 1) “Was any question unclear?” 2) “Did you have to re-read any questions and why?” and 3) “Did you perceive lead bias in any question, and if so please explain?” A total of 20 physicians participated in the focus groups. The survey was also distributed at a continuing education national conference on topics in clinical medicine with a total of 108 respondents, 11 of whom were asked the same 3 feedback questions. The survey was revised after each focus group. The study was approved by the Johns Hopkins Medicine Institutional Review Board (#NA_00079329).

### Extent of overtreatment

We asked “In your specialty, what percent of overall care do you think is unnecessary?” as well as follow up questions regarding different classes of interventions, including prescription medications, tests, and procedures. Tests were defined as diagnostic, laboratory, and radiographic.

### Reasons for overtreatment

We asked, “Nationally, what do you think are the top reasons for overutilization of resources, if any?” and “In your opinion, what can decrease overutilization?” Focusing on fee-for-service bonus pay to physicians, we asked “What do you think is the percentage of physicians who perform unnecessary procedures when they profit from them?” We also asked, “If physician compensation were to change to de-emphasize fee-for-service bonus pay, what do you think would be the impact on utilization and national health care costs?” in separate questions.

### Statistical methods

Physician perceptions on overtreatment, causes, and solutions are presented as descriptive statistics. Respondents identified percentage ranges of overutilization. Tabulated ranges are reported both as raw data and, to describe a summary response for the study population, as median responses. Median responses were estimated using spline interpolation from plots of percentage of respondents citing increasing ranges of percent overtreatment on the x axis and cumulative percentage of respondents on the y axis.[16] The chi-square test was used to compare responses on the extent of overtreatment by type of intervention. Associations between survey responses and respondent characteristics were studied using multivariate logistic and ordinal logistic regression models. Statistical analyses were performed using STATA/MP software, version 12 (StataCorp, College Station, TX).

## Results

Of the respondents, 47.9% (1,009/2,106) were attending physicians, of whom 46.0% had at least 10 years of experience (Table 1). Specialists made up 42.4% of the overall sample; the most common specialties were General Internal Medicine (40.9%), specialized Internal Medicine (10.8%), and Family Medicine (10.0%). Similarly, in 2010, the specialty with the largest numbers of active physicians in the U.S. was primary care (internal medicine and family medicine/general practice).[17, 18] Most respondents worked in hospitals that were non-profit (66.6%), academic (74.3%), and urban (67.6%).

**Fig 1:**
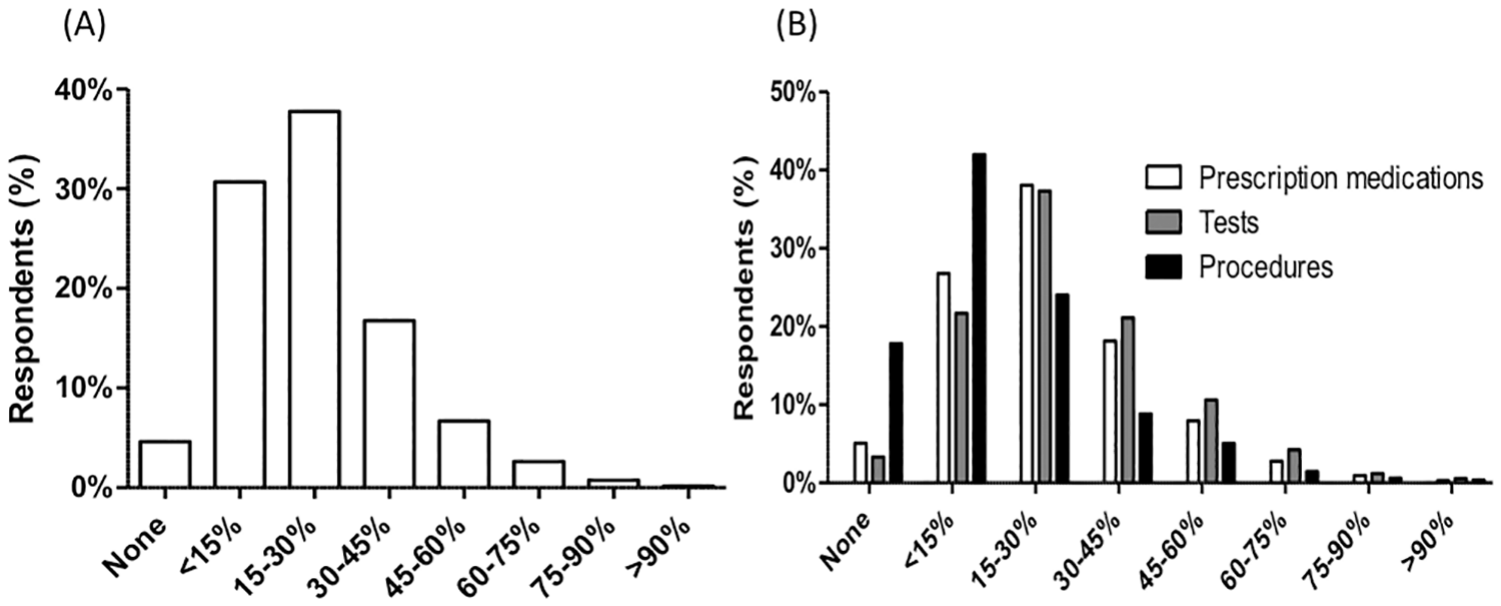
Physician perceptions on overtreatment. (A) Percentage of Overall Medical Care Considered Unnecessary (N = 2,106) (B) Percentage of Medical Care Considered Unnecessary by Type (N = 2,106).

The interpolated median response for the percentage of care delivered that is unnecessary was 20.6% for overall medical care, 22.0% for prescription medications, 24.9% for tests, and 11.1% for procedures (Fig 1A and 1B). Twenty-seven percent of respondents believed that at least 30–45% of overall medical care is unnecessary; 4.6% of respondents believed that none of the medical care delivered is unnecessary. Thirty percent believed that at least 30–45% of prescription medications are unnecessary, 37.7% believed that at least 30–45% of tests are unnecessary, and 16.2% believed that at least 30–45% of procedures are unnecessary. Eighteen percent believed that no procedures are unnecessary. Respondents believed that more tests and fewer procedures are unnecessary compared to overall medical care (p<0.001).

**Fig 2:**
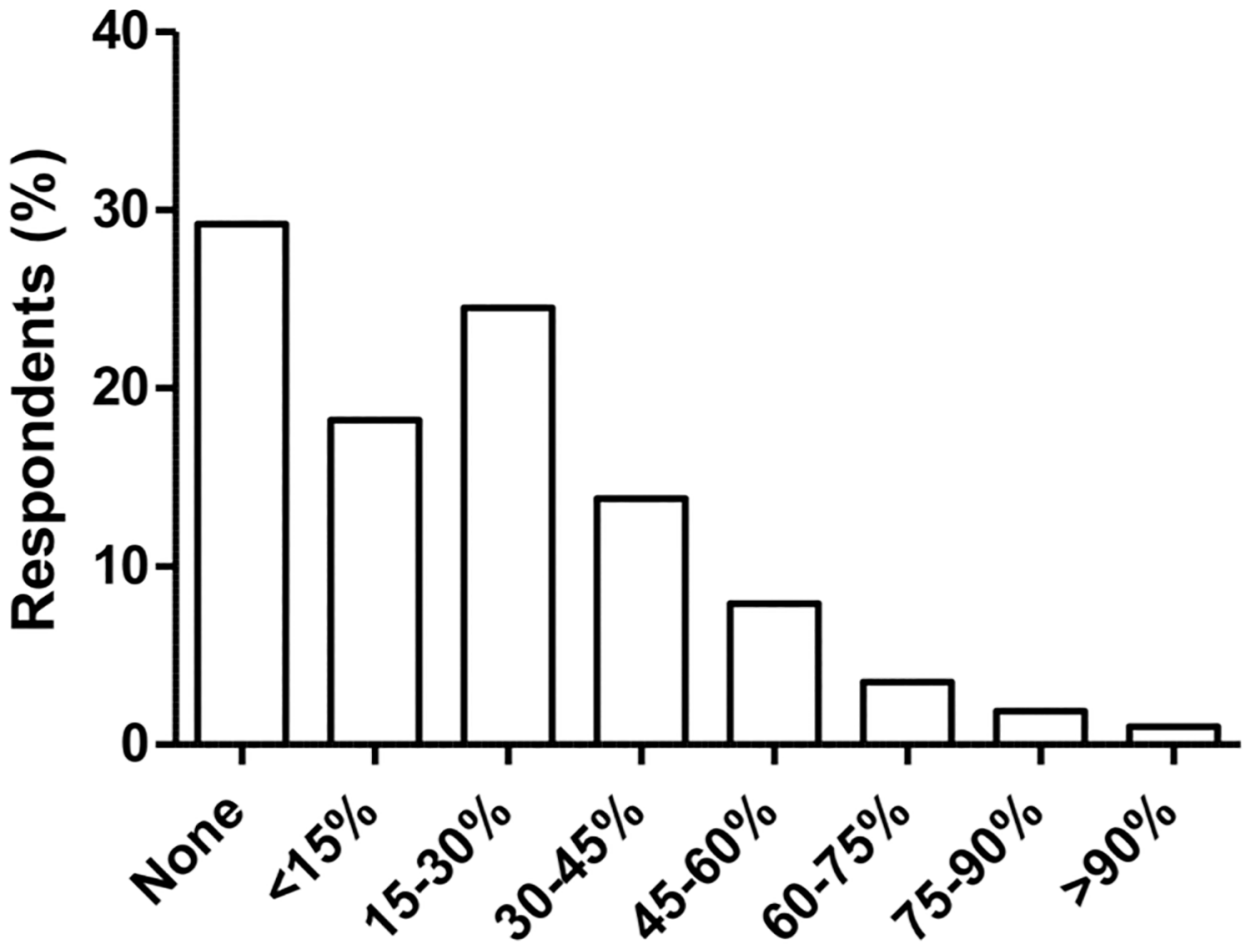
Physician perceptions on percentage of physicians who perform unnecessary procedures when they profit from them (N = 2,106).

The top three cited reasons for overtreatment were “fear of malpractice” (84.7%), “patient pressure/request” (59.0%), and “difficulty accessing prior medical records” (38.2%) (Table 2). Seventy-one percent of respondents believed that physicians are more likely to perform unnecessary procedures when they profit from them. The interpolated median response for the percentage of physicians who perform unnecessary procedures with a profit motive was 16.7%; 28.1% of respondents believed that at least 30–45% of physicians do so (Fig 2). Respondents who were attending physicians with at least 10 years of experience (OR 1.89 (1.43–2.50) vs trainees) and specialists (OR 1.29 (1.06–1.57)) were more likely to believe that physicians perform unnecessary procedures when they profit from them (Table 3). Respondents’ compensation method and hospital characteristics were not associated with differences in perceptions on the profit motive associated with unnecessary care.

**Table 2:** Physician perceptions on top three reasons for overtreatment (N = 2,106).

	Respondents	%
Fear of malpractice	1783	84.7%
Patient pressure/request	1242	59.0%
Difficulty accessing prior medical records	804	38.2%
Borderline indications	793	37.7%
Inadequate time to spend with patients	788	37.4%
Lack of adequate information/previous medical history	772	36.7%
Pressure from the institution/management	437	20.8%
Looking good in performance evaluations	282	13.4%
The hassle of communicating with other physicians	251	11.9%
Pressure from colleagues	248	11.8%
Financial security of physicians	194	9.2%
I don’t think there is any overutilization	23	1.1%

**Table 3:** Characteristics of physicians who believed that a greater number of physicians perform unnecessary procedures when they profit from them (N = 2,106).

	OR	95% CI	P-value
**Clinician characteristics**			
Level of experience			
Trainee	Ref		
Attending, less than 10 years	1.14	0.90–1.46	0.28
Attending, at least 10 years	1.89	1.43–2.50	<0.001
Male	1.13	0.93–1.38	0.22
Specialty			
Primary care	Ref		
Specialist	1.29	1.06–1.57	0.01
Type of Compensation			
Salary only	Ref		
Fee-for-service	0.86	0.70–1.06	0.16
**Hospital Characteristics**			
Hospital size			
<250 beds	Ref		
250–500 beds	0.98	0.74–1.29	0.87
>500 beds	1.00	0.74–1.35	0.99
Hospital type			
VA/Government	Ref		
For-profit	1.20	0.84–1.72	0.32
Non-profit	1.14	0.83–1.56	0.43
Type of institution			
Non-academic	Ref		
Academic	1.02	0.79–1.33	0.86
Setting			
Urban	Ref		
Suburban	0.83	0.65–1.05	0.11
Rural	0.80	0.54–1.19	0.28

The top three cited potential solutions were “training residents on appropriateness criteria” (55.2%), “easy access to outside health records” (52.0%), and “more practice guidelines” (51.5%) (Table 4). Seventy-six percent of respondents believed that de-emphasizing fee-for-service bonus pay would reduce unnecessary utilization; 70.8% believed that it would reduce national healthcare spending, with the median reduction being 10–20% (Table 5).

**Table 4:** Physician perceptions on potential solutions to overtreatment (N = 2,106).

	Respondents	%
Training residents on appropriateness criteria	1163	55.2%
Easy access to outside health records	1096	52.0%
More practice guidelines	1084	51.5%
Listing prices when ordering tests	910	43.2%
Increase base salaries and decrease fee-for service	613	29.1%
More peer review	489	23.2%
More government regulation	240	11.4%

**Table 5:** Physician perceptions on potential impact of reducing fee-for-service physician reimbursement (N = 2,106).

	Utilization		National Healthcare Costs	
	Respondents	%	Respondents	%
Decrease by >30%	235	11.2%	242	11.5%
Decrease by 20–30%	505	24.0%	434	20.6%
Decrease by 10–20%	531	25.2%	424	20.1%
Decrease by <10%	329	15.6%	392	18.6%
Stay the same	433	20.6%	522	24.8%
Increase by <10%	26	1.2%	29	1.4%
Increase by 10–20%	24	1.1%	34	1.6%
Increase by 20–30%	12	0.6%	17	0.8%
Increase by >30%	11	0.5%	12	0.6%

## Discussion

We found that most physicians surveyed (64.7%) believe that at least 15–30% of medical care is unnecessary, representing a significant opportunity to reduce waste in health care. We also found that physicians perceive fear of malpractice, patient demands, and difficulty accessing prior medical records as the most common reasons for overtreatment. To our knowledge, this is the first study to survey physicians on overtreatment in a range of specialties and on a nation-wide level. The important study by Sirovich et al only included primary care physicians, excluding doctors within procedure-heavy specialties that could contribute to overall overutilization within healthcare. Since 2011, there has been one survey study to date that focused on perceptions of low value care among 189 physicians that supports the use of evidence based initiatives such as the Choosing Wisely campaign to help raise awareness of overutilization among clinicians.[19]

However, there are some important limitations. First, we did not study the issue of undertreatment, which is another form of poor quality care that may result in unnecessary health care spending from patient complications. Second, our survey population included a large percentage of academic physicians, who may have different views about overtreatment in comparison to their peers. Third, our sample size was not large enough to compare responses across specific specialties. Fourth, we used interpolated medians to estimate the overall response to some questions, which may not represent the true average. The alternative of asking respondents to provide an exact percentage, however, may have introduced bias or been a barrier to survey completion. Fifth, there may be a response bias inherent to any solicitation of doctors to participate in a survey and overtreatment is not absolutely equivalent to unnecessary medical care, the latter of which was the subject of the survey questions. We did not single a group of doctors out, but rather used AMA masterfile listed doctors who responded to an inquiry. Despite this method, we believe that a notable strength is that the data represent the direct voice of physicians on a physician-level problem that is under-recognized, yet endemic in health care. Sixth, the data was collected in 2014 and does not capture the effect of more recent initiatives and programs that address overutilization. Nevertheless, overtreatment continues to be a major contributor to excessive healthcare spending and this data remains relevant nationally. Finally, this study was conducted in the United States but overtreatment is a global issue and continues to be cited in the literature as such. A recent article in the BMJ from London cites a list of 40 unnecessary interventions chosen by the Academy of Medical Royal Colleges modeled after the Choosing Wisely Initiative. [20]

At 85% of respondents, “fear of malpractice” was the top cited reason for overtreatment. Perceptions on the prevalence of malpractice suits, however, may be greater than the reality of the problem. Only 2–3% of patients harmed by negligence pursue litigation, of whom about half receive compensation.[21] Paid claims have declined by nearly 50% in the last decade,[22] and it has been suggested that honest disclosure and an offer of an apology by the physician can further mitigate litigation.[21] Despite this trend, in a survey of 627 primary care physicians, the top three cited reasons for overtreatment were malpractice concerns (76%), clinical performance measures (52%), and inadequate time to spend with patients (40%).[12] On the patient side, the perception that more care is better care is also a factor and can be fueled by goals to achieve high patient satisfaction scores. Studies indicate that focused patient education through shared decision making between patients and physicians results in more conservative care.[23] Patient decision aids have been associated with a 19% lower likelihood of patients receiving antibiotics for acute bronchitis or an upper respiratory infection and significant reductions in elective surgery use.[24,25] Most patients prefer to leave medical decisions to their physician,[26] suggesting that greater attention to shared decision making can be a powerful tool to reduce overtreatment. Moreover, improved data sharing can reduce the number of tests, procedures, and patient encounters, though the impact on overuse may be limited. In a study of California and Florida emergency departments, regional implementation of electronic health information exchange reduced repeat CT scans by 8.7%, ultrasounds by 9.1%, and chest X-rays by 13.0%.[27] With increased hospital consolidation in recent years, health systems should make data sharing a priority both within and among health systems. Complete interoperability of healthcare information nationwide may yield an estimated net savings of $78 billion a year.[28]

In our survey, most respondents (70.8%) believed that physicians provide unnecessary procedures when they profit from them. With regards to physician compensation, both the fee-for-service and flat salary models have been criticized for their potential unintended consequences. Many large healthcare systems, including Kaiser Permanente, Mayo Clinic, Cleveland Clinic, and MD Anderson Cancer Center have eliminated compensation schemes based on clinical throughput. On the other hand, some hospitals are moving toward bonuses more heavily-weighted for clinical volume to increase physician productivity. This view is consistent with a previous systematic review showing that fee-for-service physician reimbursement is associated with more primary care visits, referrals to specialists, and diagnostic and curative services.[29] Profit motives may manifest in other ways. For example, urology practice groups able to self-refer patients to radiation treatment are 29% more likely to prescribe radiation than non-self-referring centers.[30] Also, fee-for-service hospital compensation has been implicated by respondents as a driver of overtreatment. Payers in some areas have introduced global payment to hospitals. In Florida, payers have begun to reimburse cancer care using bundled payments. Maryland has legislated a new global payment system so that hospitals will have a financial incentive to reduce health care waste, presumably including overuse.[31] Research on integrated health systems, such as Geisinger Health System, suggests that hospital-insurer alignment on health outcomes can result in lower cost care.[32] Further evaluation of the impact of these payment schemes on overtreatment can lead to generalizable models of delivery that reduce patient harm and wasteful spending.

Addressing overtreatment can have a major impact on rising health care costs in the US. Using the IOM’s estimate of excess costs arising from overtreatment,[1] a 50% reduction in “unnecessary services” would result in $105 billion in savings each year, or about 4% of total national healthcare spending. Fragmented and outdated practice guidelines can be problematic and contribute to the problem of overdiagnosis.[33] Also, overdiagnosis can result in increased screening and unnecessary imaging and procedures. Guidelines should consider over-treatment consequences rather than simply increasing screening or diagnosing for a particular patient presentation. In seeking to raise awareness on overtreatment, the American Board of Internal Medicine launched the Choosing Wisely campaign, which has resulted in over 60 specialty “Top Five” lists being published.[34] At our own institution, more than 20 resident groups from the Departments of Surgery and Emergency Medicine have implemented quality improvement projects to address Choosing Wisely goals. A second campaign called “Improving Wisely” is now addressing overtreatment by identifying physician-endorsed metrics of overtreatment in big data and then notifying outlier physicians of their performance in a confidential, peer-to-peer manner.

Future work should focus on the most high-volume over-utilized tests and procedures by specialty. Medical school and training should include guidance on the subject of appropriateness before doctors are exposed to the factors identified in this study as factors contributing to the problem. Health care utilization by physicians may be influenced by the cost environment in which they trained as residents,[35] suggesting that early intervention may be helpful. Training on appropriateness criteria and practice guidelines should be a priority for the future. The effectiveness of these interventions warrants further study as they are not yet well known to increase appropriateness of medical care. Physician engagement is needed to address the problem of overtreatment in health care.
